# Leicester Royal Infirmary

**Published:** 1917-04-07

**Authors:** 


### LEICESTER ROYAL INFIRMARY.

### A Largely Attended Annual Meeting.

Many interesting features were presented at the 144th anniversary meeting of the Leicester Royal Infirmary, presided over by Mr. T. Fielding Johnson, jun., chairman of the board of governors.

The nurses' recreation room was well filled by a large attendance, displaying the fact that contributors to a record year's income were not content only to send in their contributions, but desired to show their interest in the administration.

The Chairman's survey of the work of the year showed that his period of many years' service as chairman of the house committee had enabled him to grasp the intricate details of administration, and present them in a way which made them pregnant with an interest which never flagged for a full hour and a half.

The essential statistics of income and expenditure were submitted in a manner which enabled them to be readily followed, and were made realistic by comparisons with the figures of 1914, being those which could most fittingly and usefully be drawn. The income proved that the town and county had participated in a remarkable way in the prosperity (fictitious though it was, being transitory) which the war had brought to some large manufacturing centres. The extraordinary income gave point to his observation. In 1914, 36 per cent. of legacies were used as income for the year, and in 1915 legacies were entirely absorbed in the general expenses, whereas in 1916 it had been possible to put all legacies into capital and thus increase the investments by £15,000; an amount counterbalanced by the writing off of nearly £17,000 for depreciation in the value of former investments. The expenditure of 1914 had been exceeded in 1916 by a 54 per cent. increase on provisions, 9 per cent. on drugs and chemicals, 20 per cent. on domestic expenditure, and 30 per cent. on salaries and wages. *Per contra* reductions of 28 per cent. were shown in establishment charges and of 11.5 per cent. in management expenses. The need for co-operation between the various alleviative agencies of the town and county was never more marked than at present, and the composition of the board, comprising as it did representatives from all the municipal and charitable agencies and interests, demonstrated that the governors were earnest in their desire that the work of the infirmary should be as helpful as it was possible to make it to the entire community. Tribute was paid to the work of the Faire Hospital and the Highfields Hospital (which were largely supported by contributions from the patients), to the nursing institutions, to the municipal hospitals, to the Saturday Hospital Society, and to the administration of the large military hospitals established in the town.

The need for extension of the massage and electro-therapeutic department by equipping an orthopædic department with Zander mechanical apparatus for physical exercises and movements had been made apparent in the treatment of military patients. The pathological department had been more firmly established by the appointment of a salaried pathologist, and the department would be of much greater service to the community now that the institution had entered into an arrangement with the local authorities for the treatment of venereal diseases under the L.G.B. scheme, a scheme which had for the first time introduced a lady doctor on the staff. The continued growth of the institution was shown by the fact that whereas a few years ago one of every three patients came from the county areas, the figures now showed that 40 per cent. of in-patients and 45 per cent. of out-patients attended from those areas.

The Mayor of Leicester ascribed the prosperity, popularity, and usefulness of the institution to the democratic administration, and expressed his willingness that the advantage of co-operation should further be shown by financial help from his £100,000 Soldiers Fund in the building and equipment of the suggested orthopædic department.

The Chairman of the sanitary committee of the Corporation alluded to the helpful way in which the County and Town Councils had been met in the arrangements for the treatment of venereal diseases now undertaken by the infirmary, and to the helpful co-operation which already existed in the treatment of zymotic diseases, arising within the infirmary, in the Corporation Hospital, and to the treatment of medical and surgical conditions from that hospital in the infirmary.

The report having been adopted, Mr. B. W. Russell (chairman of the house committee) moved a resolution expressing the regret of the governors at the attainment by Lieutenant-Colonel Pratt, the senior physician, of the age limit prescribed by the rules, which necessitated his retirement from the active staff, after thirty-one years of almost continuous service, and appointing him a consulting physician. Captain F. W. Bennett paid tribute to Colonel Pratt's influence on the medical life of the district, and Dr. C. A. Moore, house surgeon to the institution at the time of Colonel Pratt's first resident appointment, alluded to the progress which had taken place in medicine and surgery since that period.

Mrs. Howard Symington, from the County, and Mrs. Russell Frears, from the Borough, respectively proposed and seconded a resolution of thanks to the hon. medical and surgical staff for their work. On the motion of Mrs. Banton, seconded by Mrs. Riley, the election of the retiring members of the board was agreed to after speeches which bore tribute to the useful service of women as hospital administrators.

Mr. J. E. Faire, president of the hospital which bears his name, thanked the chairman for presiding, and congratulated him on his accession to the chairmanship of the Royal Infirmary.

